# The relationship between acculturative stress and psychological outcomes in international students: a systematic review and meta-analysis

**DOI:** 10.3389/fpsyg.2024.1403807

**Published:** 2024-07-03

**Authors:** Rasa Soufi Amlashi, Mohammadreza Majzoobi, Simon Forstmeier

**Affiliations:** Developmental Psychology and Clinical Psychology of the Lifespan, University of Siegen, Siegen, Germany

**Keywords:** acculturation, stress, depression, anxiety, international students, systematic review, meta-analysis

## Abstract

**Introduction:**

The current systematic review aimed to examine the relationship between acculturative stress (AS) and psychological outcomes in international students to determine the role AS may play in predicting the mental health of international students.

**Methods:**

The studies included in the current systematic review and meta-analysis had considered AS and its impact on psychological outcomes among international students studying abroad. After checking the studies found in our primary search through the scientific databases in terms of our eligibility criteria, 29 studies were included, of which 26 were eligible for a meta-analysis (total *N* = 7,247).

**Results:**

Meta-analysis indicated a moderate mean correlation of AS with psychological outcomes like depression, life satisfaction, quality of life, vocational outcome expectations, drinking behaviors, resilience, health promotion behavior, psychological adjustment, psychological distress, negative affect, and mental health symptoms (*r* =  0.39) and depression (*r* =  0.41), respectively.

**Discussion:**

The review of studies revealed a robust relationship between AS and increased negative psychological outcomes such as depression, psychological distress, and general stress, as well as decreased positive psychological outcomes such as psychological adjustment, mental health, life satisfaction, and quality of life.

## Introduction

Thanks to the advancement of technology in today’s world and the ease of moving from one country to another, it has become common for people to live in a country other than their home country. One of the groups facing this most is students. Today, it is clearly seen that a significant number of people move abroad to pursue a university education. Statistics show that in 2017, 5.3 million students traveled to a country other than their home country to continue their education, more than half of whom were studying in six countries: the United States, the United Kingdom, Australia, France, Germany and Russia (Bustamante, 2020). Moreover, in 2019 alone the number of foreign students entering the United States to study was about 1,095,299, which is 5.5% of all American university students (Bustamante, 2020). This figure for Germany (Learn German, 2020) increased significantly from 301,350 in 2013 to 394,665 in 2019. This statistic for Australia also increased from 465,508 in 2015 to 738,107 in 2019 (Study in Australia, 2019). Having better job opportunities and different experiences and being familiar with other professors’ views in more developed countries are the main motivations for people to continue their studies in another country. However, Zhou et al. (2008) stated that one of the most important challenges for students studying abroad is facing a new culture, and other studies have also shown that the most important challenge for students studying abroad is to face the new culture (Yeh and Inose, 2003; Zhou et al., 2008; Luo, 2014).

Culture in general refers to a set of ways of life of a people or a nation based on social, political and economic customs and especially refers to the unique system of national beliefs and morals of human beings (Okonta, 2020). Entering a new culture can contribute to the development of psychological problems such as depression (Bernstein et al., 2011), anxiety (Suarez-Morales and Lopez, 2009), and bulimia nervosa (Kroon Van Diest et al., 2014). Adapting to different conditions in the destination country, managing the leading problems in this new culture and finding a different balance from what was experienced in the country of origin have created a new construct in this area called acculturation (Gholamrezaei, 1995). Various studies have shown a relationship between successful acculturation and self-esteem (Gholamrezaei, 1995), better mental well-being and less depression (Miller et al., 2011; Geeraert et al., 2014; Hirai et al., 2015; Toth-Bos et al., 2020). In contrast, unsuccessful acculturation has negative consequences on individuals’ psychological and social/cultural adjustment (Searle and Ward, 1990). Acculturation, whether successful or unsuccessful, is considered as an important challenge that is inevitably associated with stress. This has led to the formation of a construct called acculturative stress (AS), which is the stress of facing those life events that stem from the experience of acculturation (Berry, 2006).

### The models of acculturative stress

One of the most prominent and original models in the field of acculturation is Berry’s (2006) AS model. According to this model, the differences between the host society and the individual’s local society, as well as the requirements of the new culture in which the individual intends to live, are a crucial challenge for immigrants. If a person evaluates this challenge as a serious crisis and doubts his ability to manage it, he may experience AS, and the longer this stress lasts, the more mental resources a person needs to deal with, and in an erosive process, it leads to the creation of psychological disorders such as anxiety, depression, and psychosomatic disorders. Finally, the model states that if a person has a successful socio-cultural adaptation to his host society in a long-term process, he will also experience a suitable psychological adaptation.

The models represented regarding the acculturation stress are usually classified in three categories of psychopathology, stress and coping, and culture learning/social skills (Ward, 2008). Comparing these models, Ward (2008) argued that Berry’s model, by focusing on three elements of cultural learning, stress, and coping offers a more positive and adaptive approach compared to earlier perspectives like Oberg’s (1960) culture shock theory, which highlighted emotional distress, shock, and anxiety in the acculturation process. Ward posits that Berry’s model is beneficial for two reasons: first, any change in life, whether positive or negative, induces stress and triggers coping strategies; and second, the use of coping styles may either be beneficial, leading to adaptive outcomes, or unsatisfactory, resulting in maladaptive and pathological consequences.

However, Ward (1996) contends that in addition to psychological adaptation, sociocultural adaptation, specifically social skills, should also be considered. Although these two types of adaptation may interact, they also have distinct differences. Psychological adaptation is defined based on concepts such as psychological well-being and satisfaction, viewed within the framework of stress and coping styles, and is significantly influenced by personality, social support, and life changes. In contrast, sociocultural adaptation, which is defined by variables such as skill deficits and social difficulties and viewed from a cultural learning perspective, is generally influenced by factors such as the length of residence in the host society, prior experiences of intercultural relocation, and the extent of interaction with the host culture (Ward, 1996). In other words, culture learning/social skills emphasize behavioral skills (social inadequacies) instead of affective and health outcomes (psychological inadequacies), stating that international students face difficulties because of trouble negotiating everyday social situations, learning second culture and using social skills.

The final stage in Berry’s (2006) AS model refers to the adaptation to the host society over time. In other words, the adaptation process is a time-consuming process, and in the middle of this process, people may suffer from the psychological consequences of AS. It is obvious that people can tolerate an optimal level of stress and when this stress becomes a long-term process, it may result in negative psychological consequences. Therefore, the theory underlying this review article assumes that people are thought to be vulnerable to psychological disorders during the acculturation process from the point they experience AS until they reach a relatively complete adaptation to the host culture. We assume that “time” is a very decisive factor in the acculturation process, and the length of time people live in the host society plays a highly determinative role in their mental health. We speculate that the process of acculturation, like other stressful processes, can be described as an inverted U curve. In other words, in the first years of the process of acculturation, people experience a high level of stress, and as time passes and they approach the point of relative adaptation, their stress returns to the optimal level. Therefore, *time* as a variable that is placed on the horizontal axis of the inverted U diagram has a decisive role in reducing stress. According to the Selye’s (1956) general adaptation syndrome (GAS) model, when the stress is higher than the optimal level, it triggers the fight-or-flight response. The prolongation of the person’s resistance process in stressful situations brings him to the stage of exhaustion, in which the person is enough vulnerable to suffer from psychological disorders. Therefore, the first years of immigration may be years full of psychological damage, and time can moderate these damages. Consistent with the mentioned theory, in recent years, the relationship between AS and important psychological variables such as depression (Revollo et al., 2011; Cho et al., 2018), anxiety and homesickness (Revollo et al., 2011) have been proved.

### Previous reviews and meta-analyses

There are found a number of systematic reviews and meta-analyses regarding AS and its psychological outcomes. For instance, Bridges et al. (2021) conducted a systematic review and meta-analysis in order to examine the relationship of acculturation and depression in Latinx adults. Gonzalez-Guarda et al. (2021) considered the relationship between AS and physical health consequences among Latinx individuals in the United States through conducting a systematic review. However, none of them has been conducted in international students. We found only one systematic review conducted in international students in which they examined the efficacy of the psychoeducational, cultural orientation, socio-cultural interventions in reducing AS in international students worldwide (Aljaberi et al., 2021). Therefore, there are not found any systematic review to consider the relationship between AS and psychological outcomes in international students.

### The aim of this systematic review

Although there have been many studies considering the correlation of acculturation and its related stress have been conducted with the above-mentioned important psychological variables, and even systematic reviews (Alemu and Cordier, 2017; Alidu and Grunfeld, 2018; Choy et al., 2021) have been done in this area, there is a lack of an integrated and codified message on the relationship between AS and its psychological outcomes for international students. In addition, according to the importance of international students’ psychological status in predicting their success in achieving their goals, the study of variables which seem to play a crucial role in predicting their mental status may have worthy implications for researchers, therapists, consolers and even authorities and policy makers working in this field. Therefore, the current systematic review and meta-analysis seeks to provide a pluralization in this regard. The first aim was to synthesize existing studies on the relationship between AS and its psychological outcomes in international students. Likewise, the second purpose was to determine the effect size of the relationship between AS and its psychological outcomes in international students.

## Methods

### Pre-registration of review protocol

The protocol of the systematic review and meta-analysis has been registered on the Open Science Framework (OSF) website.^1^

### Search strategy

**Figure 1:**
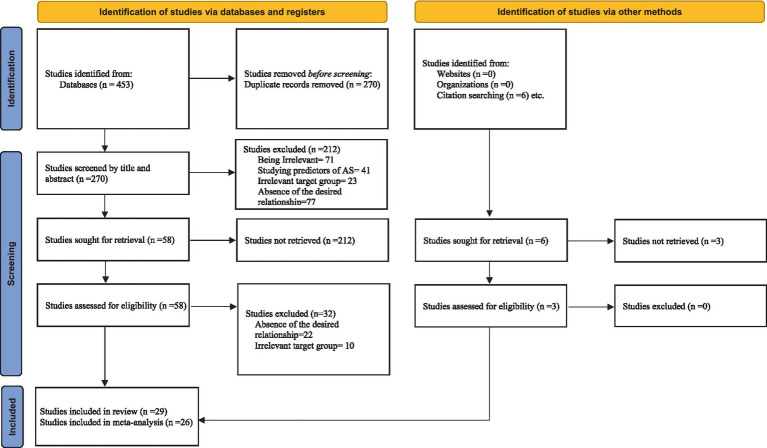
Flowchart of studies found through literature search and screening (Page et al., 2021).

To find eligible studies to include in this systematic review and meta-analysis, we used our search query to search in scientific databases, including EBSCOhost (APA, PsycArticles, Psychology and behavioral sciences collection, PSYNDEX literature with PSYNDEX tests), Proquest (Social Sciences), Wiley Online Library, PubMed, OVID, and Web of Science. Our search query was (“international student*” OR “foreign student*” OR “sojourn*”) AND (“accultur*” OR “culture*”) AND (“psycho*” OR “mental*” OR “self*” OR “cognit*” OR “emot*” OR “behave*” OR “health*” OR “well-being” OR “adjust*” OR “Adapt*” OR “resilience” OR “Quality” OR “social*” OR “depress*” OR “stress*” OR “anxiety” OR “lonel*” OR “symptom*” OR “drink*” OR “distress” OR “homesickness” OR “life*”). The titles of the articles found using this search query were reviewed by the first author to determine whether they met the criteria to be included in the stage of exact screening of the title and abstract. The selected articles were then reviewed by the second author and an expert in this field, and finally the articles that remained for screening the title and abstract were uploaded in Eppi-Reviewer online software, where both authors could review the existing articles based on eligible criteria. In the first stage in the above-mentioned software, the title and abstract of the articles were reviewed, and then in the second stage, the full-text of the remaining articles was evaluated based on our inclusion and exclusion criteria to determine the final articles suitable for this systematic review. To ensure the literature saturation, a manual search was performed in the references list of residual studies in the full text review phase. Flowchart of studies found through literature search and screening is presented in Figure 1.

### Inclusion and exclusion criteria

We looked for studies that have generally examined the relationship between AS (and its related variables such as acculturation, cultural shock and suchlike) and one or more psychological outcomes such as depression, psychological distress, alcohol abuse, mental health symptoms, negative emotions, perceived general stress, premenstrual stress, psychological adjustment, mental health, career outcome expectation, life satisfaction, health-promoting behaviors, quality of life, resilience, a sense of coherence so on in international students. To be included in our systematic review, studies had to (1) be written in English, (2) be published between 1980 and 2020 in a peer-reviewed scientific journal, (3) be conducted on the general and healthy human community studying at a university located in a country other than the home country, (4) study individuals without cognitive impairments or some form of disability, and (5) study heterosexual individuals. Studies conducted on refugees studying in a country other than their home country were excluded. There were no restrictions on the geographical location in which the study was conducted.

### Risk of bias

**Figure 2:**
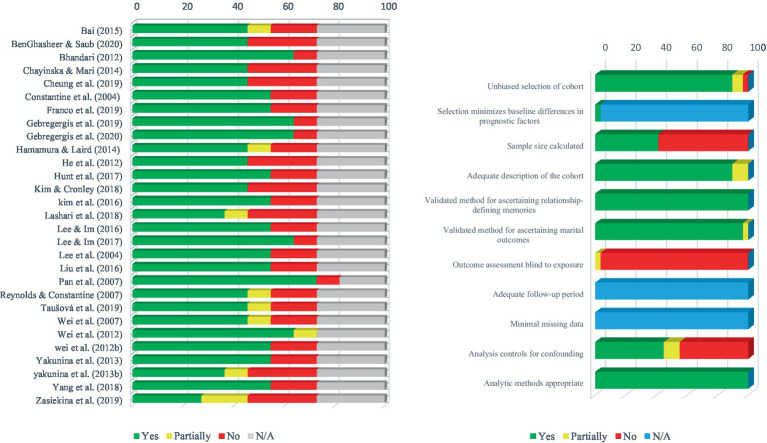
Risk of bias assessment in terms of studies as well as criteria presented in the ARHQ assessment tool.

To estimate the risk of bias in the studies included each of these studies was considered by the first author (RA), the second author (SF) and another independent person expert in the field of inquiry. We also used the Agency for Research and Healthcare Quality Scale (AHRQ) as a tool to examine the bias of studies included. This measurement tool is compatible with various study designs (Williams et al., 2010; Taylor et al., 2015; Forrester et al., 2017; Majzoobi and Forstmeier, 2022). This tool contains 11 criteria for assessing the quality of studies, each of which also has several subscales, to assess important methodological factors of a study. The criteria of this scale included unbiased selection of the cohort, selection of minimized baseline differences in prognostic factors, sample size calculated to be at 5% difference, adequate description of the cohort, validated method for ascertaining exposure, validated method for ascertaining clinical outcomes, outcome assessment blind to exposure, adequate follow-up period, completeness of follow-up, analysis controls for confounding, and appropriate analytic methods. The methodology and results of the input studies are reviewed according to the above criteria to determine whether they meet these criteria. Depending on whether they “fully,” “to some extent” and “not at all” meet the criteria, one of the words “No,” “Yes” and “Partially” is assigned to each study for each criterion. If a study does not meet the required quality or the necessary methodological criteria mentioned in this tool, it is better to consider it as a probably biased article and exclude it from the review. Using this tool, we rated 11 criteria concerning each individual paper via “Yes,” “No,” “partial,” or “cannot tell” terms. The results can be seen in Table 1. In addition, Figure 2 represents a visual representation of bias within and across studies.

**Table 1:** Risk of bias assessment of studies included based on the Agency for Research and Healthcare Quality assessment tool (Williams et al., 2010).

Authors	Unbiased selection of cohort	Selection minimizes baseline differences in prognostic factors	Sample size calculated	Adequate description of the cohort	Validated method for ascertaining AS^a^	Validated method for ascertaining PC^b^	Outcome assessment blind to exposure	Adequate follow-up period	Minimal missing data	Analysis controls for confounding	Analytic methods appropriate
Bai (2016)	Yes	N/A	No	Yes	Yes	Partially	No	N/A	N/A	Yes	Yes
BenGhasheer and Saub (2020)	Yes	N/A	No	Yes	Yes	Yes	No	N/A	N/A	No	Yes
Bhandari (2012)	Yes	N/A	Yes	Yes	Yes	Yes	No	N/A	N/A	Yes	Yes
Chayinska and Mari (2014)	Yes	N/A	No	Yes	Yes	Yes	No	N/A	N/A	No	Yes
Cheung et al. (2020)	Yes	N/A	No	Yes	Yes	Yes	No	N/A	N/A	No	Yes
Constantine et al. (2004)	Yes	N/A	No	Yes	Yes	Yes	No	N/A	N/A	Yes	Yes
Franco et al. (2019)	Yes	N/A	No	Yes	Yes	Yes	No	N/A	N/A	Yes	Yes
Gebregergis et al. (2019)	Yes	N/A	Yes	Yes	Yes	Yes	No	N/A	N/A	Yes	Yes
Gebregergis et al. (2020)	Yes	N/A	Yes	Yes	Yes	Yes	No	N/A	N/A	Yes	Yes
Hamamura and Laird (2014)	Partially	N/A	Yes	Yes	Yes	Yes	No	N/A	N/A	No	Yes
He et al. (2012)	Yes	N/A	No	Yes	Yes	Yes	No	N/A	N/A	No	Yes
Hunt et al. (2017)	Yes	N/A	Yes	Yes	Yes	Yes	No	N/A	N/A	No	Yes
Kim and Cronley (2018)	Yes	N/A	No	Yes	Yes	Yes	No	N/A	N/A	No	Yes
Kim and Yoo (2016)	Yes	N/A	Yes	Yes	Yes	Yes	No	N/A	N/A	No	Yes
Lashari et al. (2018)	Yes	N/A	No	Partially	Yes	Yes	No	N/A	N/A	No	Yes
Lee and Im (2016)	Yes	N/A	Yes	Yes	Yes	Yes	No	N/A	N/A	No	Yes
Lee and Im (2017)	Yes	N/A	Yes	Yes	Yes	Yes	No	N/A	N/A	Yes	Yes
Lee et al. (2004)	Yes	N/A	No	Yes	Yes	Yes	No	N/A	N/A	Yes	Yes
Liu et al. (2016)	Yes	N/A	No	Yes	Yes	Yes	No	N/A	N/A	Yes	Yes
Pan et al. (2007)	Yes	Yes	Yes	Yes	Yes	Yes	No	N/A	N/A	Yes	Yes
Reynolds and Constantine (2007)	Yes	N/A	No	Yes	Yes	Yes	No	N/A	N/A	Partially	Yes
Taušová et al. (2019)	Partially	N/A	No	Yes	Yes	Yes	No	N/A	N/A	Yes	Yes
Wei et al. (2007)	Yes	N/A	No	Yes	Yes	Yes	No	N/A	N/A	Partially	Yes
Wei et al. (2012a,b)	Yes	N/A	Yes	Yes	Yes	Yes	Partially	N/A	N/A	Yes	Yes
Wei et al. (2012b)	Yes	N/A	Yes	Yes	Yes	Yes	No	N/A	N/A	No	Yes
Yakunina et al. (2013a,b)	Yes	N/A	Yes	Yes	Yes	Yes	No	N/A	N/A	No	Yes
Yakunina et al. (2013b)	Yes	N/A	No	Partially	Yes	Yes	No	N/A	N/A	No	Yes
Yang et al. (2018)	Yes	N/A	No	Yes	Yes	Yes	No	N/A	N/A	Yes	Yes
Zasiekina and Zhuravlova (2019)	No	N/A	No	Partially	Yes	Yes	No	N/A	N/A	Partially	Yes

a Acculturation stress.

b Psychological consequences.

### Meta-analysis

Among the 29 studies included in this systematic review, 26 studies (total *N* = 7,247) in which the relationship between AS and a psychological outcome was studied were included in the meta-analysis. As in some included studies, the relationship between AS and more than one psychological consequence was examined, and in some studies the mentioned relationship was examined more than once. Our meta-analysis inputs were more than 26 cases and finally reached 34 cases. It should also be noted that the variable of depression, as one of the most important psychological consequences of AS, has been studied in many studies and we were able to perform a separate meta-analysis with 13 inputs to obtain the effect size of AS on depression. Therefore, a separate meta-analysis was performed to investigate this relationship. Pearson’s correlation index was used to investigate the relationship between predictor and dependent variables and this index was extracted from the included studies. Cochran’s heterogeneity test, which provides both *I*^2^ and *Q* indices to evaluate the homogeneity of effect sizes, was also used. The *I*^2^ report is mainly due to the fact that this index can provide better information compared to *Q* when the number of studies included in the meta-analysis is relatively low. One of the important assumptions of meta-analysis is the publication bias index, according to which all studies included in the analysis should have an effect size around the mean. In the present meta-analysis, funnel diagram, Egger’s regression index and Kendall’s *S* were used to test the above assumption. It is worth mentioning that in some studies instead of Pearson’s *r*, Beta and Eta values had been reported and as Beta was between 0.5 and −0.5, an online calculator^2^ was used to convert them into Pearson’s *r*. The online calculator has been designed based on the equation of *r* = 0.98*β* + 0.05*λ* by Peterson and Brown (2005). Having validated the efficacy of the equation through using data gained from 200 studies, Peterson and Brown concluded that the equation has sufficient robustness, particularly when the value of *r* and *β* are in a range from 0.5 to 0.5. The value of *λ* equals one when *β* is not negative and zero when *β* is negative. Given that the statistical samples of studies included in this meta-analysis consisted of students from different countries with different races and religious orientations, levels of education and living conditions, we used a random model, based on the advice of Hunter and Schmidt (2004, as cited in Field and Gillett 2010) to report the overall effect size in our meta-analysis. Fixed-effects models assume that the same population value (e.g., *r* or 𝒹) underlies all studies, while random-effects models allow for the possibility that these population parameters vary from study to study. Fixed-effects models only consider simple sampling error, estimated by the sampling error variance formula, ignoring sampling error variance created by variation across studies in the underlying population values. In contrast, random-effects models consider both simple sampling error and the variance created by differences in population parameters across studies. Fixed-effects models tend to underestimate the sampling error variance and the standard error of the mean, leading to narrower confidence intervals. However, random-effects models provide more accurate estimates by incorporating the variability across studies (Schmidt et al., 2009). Therefore, despite the assumption of homogeneity in the current meta-analysis, using a random-effects model is preferable. Random-effects models offer a more realistic and comprehensive consideration of error sources and study differences, ensuring more accurate and reliable results. Many experts believe that there is always some variation in population parameter values across studies due to theoretical or substantive reasons. They argue that real moderator variables cause differences in *r* and 𝒹 values between studies. However, some evidence shows that certain study areas are homogeneous at the level of population parameters (this means that population parameters stay the same when sampling error, measurement error, and range variation are accounted for), but this homogeneity can only be detected using random-effects models, which estimate the level of variability. Fixed-effects models cannot do this because they assume homogeneity from the beginning (Schmidt et al., 2009).

Doing so allows us to more confidently generalize the effect size obtained from the meta-analysis to not only the studies included in our systematic review, but to all studies conducted in this area of research (Field and Gillett, 2010). Comprehensive Meta-Analysis (CMA-2) software was used for meta-analysis.

## Results

The characteristics of the studies included in this systematic review and meta-analysis are fully described in Table 2. Accordingly, all studies included were published between 2004 and 2020. Of the total studies, 15 were conducted in the United States, four in China, two in South Korea, two in Malaysia, two in Hong Kong, and one in Italy, Australia, the Netherlands, and Ukraine. Out of the studies included, 25 had a correlational design, two were comparative studies and two had a longitudinal design. Descriptive design of most studies included has allowed us to present more consistent and integrated results. The uniformity of the design of the studies included also facilitated meta-analysis. The statistical population in these studies included international male and female students studying in a country other than their own. The lowest and highest mean ages of participants in the studies included were 19.7 to 31 years, respectively. The minimum and maximum duration of their stay in the foreign country was between 1 month and 5 years. Additional information about the characteristics of the participants can be seen in Table 2.

**Table 2:** Description of studies included.

Author, year, country	Design	Description of participants	Acculturation stress measure	Psychological outcome measure	Key outcome (effect size, if presented)
Bai (2016), USA	Correlational	Chinese international students and visiting scholars in U.S.A (*n* = 267); *M*_age_ = 26 (SD = 4.04); *M*_LOS_ = 35 M (SD = 28,09)	ASSCS (Bai, 2016)	Self-Rating Depression Scale (Zung, 1965)	AS was a significant predictor of depression (*β* = 0.490). AS was a significant predictor of life satisfaction (*β* = 0.505)
BenGhasheer and Saub (2020), Malaysia	Correlational	International students in Malaysia (*n* = 312); 111 (35.6%) females and 201 (64.4%) males; *M*_age_ = 33.6 (SD = 7.01); 119 (38.1%) Single, 191 (61.2%) Married, 2 (0.6%) Divorced/widowed; 202 (64.7%) Arabic Countries, 77 (24.67%) Asian Countries, 32, (10.2%) African Countries, 1 (0.3) USA; *M*_LOS_: 76 (24.4%) 6 M–1 Y, 110 (35.3%) 1–3 Y, 126 (40.4%) >3 Y	ASSIS-36 (Sandhu and Asrabadi, 1994)	OHRQoL (Adulyanon and Sheiham, 1997)	AS was correlated with OHRQoL significantly (*r* = 0.2, *β* = 0.205)
Bhandari (2012), South Korea	Correlational	Nepalese international students in South Korea (*n* = 130); 103 (79.2%) Male, 27 (20.8%) Female; Age: 65 (50%) with 20–29, 53 (40.8%) with 30–39, 12 (9.2%) with 40–50; 57 (43.8%) Single, 73 (56.2%) Married; *M*_LOS_: 13 (10%) <1 Y, 68 (52.3%) 1–3 Y, 34 (26.2%) 3–5 Y, 14 (10.8%) >5	ASSIS-36	MOS (SF-12) (Quality Metric I, 2010)	AS was correlated with MCS significantly (*r* = −0.362)
Chayinska and Mari (2014), Italy	Correlational	International students in Italy (*n* = 144); 96 (66.7%) females and 48 (33.3%) males; *M*_age_ = 24.2 (SD = 3.6); *M*_LOS_ = 34.22 M (SD = 26.77); 62.5% White, 18.1% Asian, 11.8% Hispanic/Latino, 2.8% Black/African, and 4.8% other; 25% European Union’s (EU) countries; 38.9% other European countries; 20.8% Asian countries (7.6% of the whole sample); 11.8% American countries; 3.5% African countries	ASSIS-36	PANAS (Mackinnon et al., 1999)	The subscales of AS included POH (*r* = −0.36), Homesickness (*r* = −0.34), PIA (*r* = −0.21), Insecurity and Fear (*r* = −48), Perceived Discrimination (*r* = −32) were correlated with participant’s affects
Cheung et al. (2020), Hong Kong	Correlational	Female international university students in Hong Kong (*n* = 154); *M*_age_ = 21.10 (SD = 2.13, 18–27); *M*_LOS_ = 1.49 Y (SD = 1.48)	MASI (Rodriguez et al., 2002)	PHQ-9 (Kroenke and Spitzer, 2002)	AS was correlated significantly with depression in time 1 (*r* = 0.18), time 2 (*r* = 0.38) and time 3 (*r* = 0.41)
Constantine et al. (2004), USA	Correlational	International college students in USA (*n* = 320); 190 (59.4%) women and 130 (40.6%) men; *M*_age_ = 23.63, (SD = 4.73, 17–51); 81 (25.3%) from African countries, 136 (42.5%) from Asian countries and 103 (32.2%) from Latin American countries	ASSIS-36	CES-D (Radloff, 1977)	AS was correlated significantly with depression (*r* = 0.69)
Franco et al. (2019), USA	Correlational	International university students in USA (*n* = 555); 257 (46.3%) men and 298 (53.7%) women. *M*_age_ = 26.35 (SD = 5.18, 18–50); 295 (53.2%) Asian/Pacific Islander, 138 (24.9%) White/non-Latino/a, 57 (10.3%) Latino/a, 29 (5.2%); Middle Eastern, 25 (4.5%) Black/African and 11 (2.0%) other; 419 (75.5%) single, 135 (24.3%) Married, 1 (2%) Divorced; *M*_LOS_ = 3.1 Y (SD = 2.49, 3 M–15 Y)	ASSIS-36	VOER (McWhirter and Metheny, 2009)	AS was correlated significantly with vocational outcome expectations (*r* = −0.31)
Gebregergis et al. (2019), China	Correlational	International university students in China (*n* = 506); 56% men and 44% women; *M*_age_ = 27.32; *M*_LOS_ = 21 M; 70% Single and 30% Married; 45% Asia, 41% Africa and 14% other	ASSIS-36	CES-D	AS was correlated significantly with depression (*r* = 0.37, *β* = 0.28)
Gebregergis et al. (2020), China	Correlational	International university students in China (*n* = 506); 284 (56%) men and 220 (44%) women; *M*_age_ = 27.32 (SD = 5.9, 17–48); *M*_LOS_ = 21.7 M (SD = 21.29, 1–120 M); 352 (70%) Single and 152 (30%) Married; 225 (45%) Asia, 203 (41%) Africa, 32 (6%) Europe, 14 (3%) Oceania, 13 (3%) Latin America, 10 (2%) North America	ASSIS-36	CES-D	AS was correlated significantly with depression (*r* = 0.37, *β* = 0.22)
Hamamura and Laird (2014), USA	Comparative	52 East Asian international students and 126 domestic students (*n* = 178); 28.1% men and 71.9% women; *M*_age_ = 21.6 (SD = 3.5, 18–46)	ASSIS-36	CES-D	AS predicted the level of depression among international students (*R*^2^ = 0.16)
He et al. (2012), Australia	Correlational	International university students in Australia (*n* = 119); 11 (9.2%) Men and 108 (90.8%) Women; *M*_age_ = 25.3, 66 (55.4%) <24 Y, 27 (22.7%) 25–29 Y and 26 (21.9%) >30 Y; *M*_LOS_ = 42.9% third-year, 25.2% second year, and 31.8% first-year students	ASSIS-36	SOC (Antonovsky, 1987)	AS was correlated significantly with SOC (*r* = −0.408)
Hunt et al. (2017), USA	Correlational	International university students in USA (*n* = 175); 43.4% women 56% men; *M*_age_ = 26.9 (SD = 6.3); 42.9% China, 10.9% India, 5.1% South Korea, 2.9% Vietnam, 2.1% Brazil, 1.7% Iran, 33.4% other	ASSIS-36	BYAACQ (Kahler et al., 2005)	AS was not correlated with alcohol use (*r* = −0.12) and was correlated significantly with negative alcohol-related consequences (*r* = 0.20)
Kim and Cronley (2018), USA	Correlational	International university students in USA (*n* = 322); 143 (44.4%) women, 179 (55.6%) men; Age = 223 (69.3%) 18–25 Y, 73 (22.7%) 26–30 Y, 18 (5.6) 31–35 Y, 8 (2.5%) 36–40 Y; 275 (85.4%) never married and 59 (18.2%) other; 121 (37.6%) India, 76 (23.6%) China, 50 (15.5%) South Korea, 20 (6.2%) Taiwan and 55 (17.1%) other; *M*_LOS_ = 115 (35.7%) <6 M, 60 (18.6%) 6 M–1 Y, 53 (16.5%) 1 Y–2 Y, 94 (29.2%) >2 Y	ILS (Yang and Clum, 1995)	MTF (Johnston et al., 2014)	AS was correlated significantly with resilience (*β* = 0.321, *p* < 0.001) and mental health (*β* = 0.594, *p* < 0.001), and was not correlated with Binge Drinking (*β* = 0.321, *p* = 092)
Kim and Yoo (2016), South Korea	Correlational	Chinese international students in South Korea (*n* = 272); 180 (66.2%) women and 92 (33.8%) men; Age = 8 (2.9%) <21 Y, 88 (32.4%) 22–24 Y and 112 (41.2%) 25–27 Y, 50 (18.4%) 28–30 Y and 14 (5.1%) >31 Y; *M*_LOS_ = 26 (9.6%) 6–12 M, 108 (39.7%) 13–36 M, 85 (31.3%) 37–60 M, 53 (19.5%) >61 M	ASSIS 20 (Yang et al., 2007)	MHPLP (Seo, 1996)	AS was correlated significantly with health promotion behavior (*r* = −0.29, *β* = −0.15)
Lashari et al. (2018), Malaysia	Correlational	International university students in Malaysia (*n* = 200); 55% men and 45% women; *M*_age_ = 30 (SD = 7.07, 22–45)	ASSIS-36	SACQ (Baker and Siryk, 1989)	AS was correlated significantly with Psychological adjustment (*R*^2^ = 0.08, *β* = −0.16)
Lee and Im (2016), USA	Longitudinal causal-comparative	Korean international students and Korean domestic students in USA (*n* = 187); *M*_age_ = 26.15 (4.22%), 64 (65.3%) single, 11 (11.2%) Married, 1 (1%) Divorced/separated/no longer partnered and 22 (22.4%) Partnered; *M*_LOS_ <60 M	ASSIS-36	PHQ-9; MDQ (Moos, 2010)	AS was correlated significantly with depressive symptoms (*β* = 0.07) and PMS (*r* = 0.48)
Lee and Im (2017), USA	Longitudinal causal-comparative	Korean international students and Korean domestic students in USA (*n* = 187), *M*_age_ = 26.15 (4.22%); 64 (65.3%) single; *M*_LOS_ <60 M	ASSIS-36	MDQ	AS was correlated significantly with PMS (*β* = 0.35)
Lee et al. (2004), USA	Correlational	Korean international students in USA (*n* = 74); *M*_age_ = 30 (19–41 Y), 52 (70%) men and 22 (30%) women; *M*_LOS_ = 31 M, 34% <1 Y, two-thirds <3 Y; 37 (50%) Single, 35 (47.3%) Married and 2 (2.7%) divorced or widowed	ILS	BSI (Derogatis and Melisartos, 1983)	AS was correlated significantly with psychological distress (*r* = 0.56)
Liu et al. (2016), China	Correlational	International university students in China (*n* = 567), (40.7%) women, (59.3%) men; (40.4%) from Africa, (43.84%) from Asia and less than 20% from Europe, North or South America and Oceania, 90% unmarried; *M*_LOS_ = 14.45 months	ASSIS-36	CES-D	AS was correlated significantly with depression (*r* = 0.46)
Pan et al. (2007), Hong Kong	Comparative	Chinese international students in Australia and Hong Kong (*n* = 627, 400 Hong Kong & 227 Australia); Hong Kong: 200 (50%) women, 200 (50%) men, Age = 72 (18%) <23 Y, 269 (76.3%) 24–30 Y and 59 (14.8%) >30 Y; 299 (74.8%) single, 97 (24.3%) Married, 4 (1%) other; *M*_LOS_ = 155 (38.8%) <0.5 Y, 27 (6.8%) 0.5–1 Y, 79 (19.8%) 1–2 Y, 72 (18%) 2–3 Y, 67 (16.8%) >3 Y; Australia: 152 (57%) women, 75 (33%) men; Age = 143 (63%) <23 Y, 78 (34.34%) 24–30 Y and 6 (2.6%) >30 Y; 210 (92.5%) single, 13 (5.7%) Married, 4 (1.8%) other; *M*_LOS_ = 43 (18.9%) <0.5 Y, 28 (12.3%) 0.5–1 Y, 47 (20.7%) 1–2 Y, 40 (17.6%) 2–3 Y, 69 (30.4%) >3 Y.	ASSCS (Pan et al., 2007)	CAS (Hamid and Cheng, 1996)	AS was correlated significantly with negative affect in Chinese international students in Australia (*r* = 0.366) and Hong Kong (*r* = 0.443)
Reynolds and Constantine (2007), USA	Correlational	International university students in USA (*n* = 261), 136 (52.1%) women, 125 (47.9%) men; *M*_age_ = 19.7 (SD = 1.74, 18–25 Y); 48 (18.4%) from Africa, 98 (37.5%) from Asia and 115 (44.1%) from Latin America	CADC (Sodowsky and Lai, 1997)	CAS_1_ (O’Brien, 1992)	Acculturative distress was significantly negatively predictive of college students’ career outcome expectations (*η*^2^ = 0.02)
Taušová et al. (2019), Netherlands	Correlational	International university students in Netherlands (*n* = 319), 162 women, 157 men; *M*_age_ = 24.51 (SD = 3.57, 17–37 Y); *M*_LOS_ = 18.49 M (SD = 13.93)	ASSIS-36	BSI; SWLS (Diener et al., 1985)	AS was correlated significantly with mental health symptoms (anxiety, depression, and somatization), (*r* = 0.46) and satisfaction with life (*r* = −0.41)
Wei et al. (2007), USA	Correlational	Chinese international students from China and Taiwan (*n* = 189), 96 (51%) women, 92 (49%) men; *M*_age_ = 27.97 (SD = 4.65); 135 (71.4%) from China, 43 (22.8%) from Taiwan and 11 (5.8) not available, *M*_LOS_ = 2.86 Y (SD = 1.98), (38%) single, (48%) married, 1 (1%) Divorced/separated/no longer partnered and (10%) in a dating relationship	ASSIS-36	CES-D	AS was correlated significantly with depression (*r* = 0.60)
Wei et al. (2012a,b), USA	Correlational	Chinese international students in USA (*n* = 188), 94 (51%) women, 92 (49%) men; *M*_age_ = 26.6 (SD = 4.4, 18–39 Y); 166 (88%) from China/Hong Kong, 21 (11%) from Taiwan, *M*_LOS_ = 2.6 Y (SD = 2), Two thirds married or dating, one third single	ASSIS-36	HSC (Green et al., 1988)	AS was correlated significantly with psychological distress (*r* = 0.50)
Wei et al. (2012b), USA	Correlational	International students in USA (*n* = 143), (52%) women, (47%) men; *M*_age_ = 28.03 (SD = 4.44, 22–42 Y); (69%) from China, (13%) from Taiwan, (18%) South Korea, *M*_LOS_ = 2.96 Y (SD = 2.38)	ASSIS-36	PSS (Cohen et al., 1983); OQ (Lambert et al., 2005)	AS was correlated significantly with psychological distress (*r* = 0.44) and perceived general stress (*r* = 0.40)
Yakunina et al. (2013a,b), USA	Correlational	International students in USA (*n* = 336), 169 (51%) women, 165 (49%) men; *M*_age_ = 25.1 (SD = 4.78, 18–46 Y); 219 (65%) from Asia, 45 (13%) from South and Central America, 38 (11%) from Europe, 19 (6%) from the Middle East, 7 (2%) from Africa, 5 (1.5%) from North America, 2 (0.6%) from the Caribbean, 1 (0.1%) from Australia and Oceania, *M*_LOS_ = 30.6 (SD = 22.44, 1–120 M)	ASSIS-36	SOS-10 (Blais et al., 1999)	AS was correlated significantly with psychological adjustment (*r* = −0.44)
Yakunina et al. (2013b), USA	Correlational	International students in USA (*n* = 386), (52%) women, (48%) men; *M*_age_ = 24 (SD = 4, 18–43 Y); (59%) from Asia, (16%) Europe and the former Union of Soviet Socialist Republics, (11%) Middle East, (8%) Latin America, (4%) North America, (2%) Australia, (0.2%) Africa (0.2%)	ASSIS-36	SOS-10	AS was correlated significantly with psychological adjustment (*r* = −0.40)
Yang et al. (2018), China	Correlational	International students in China (*n* = 299), 156 (52.17%) women, 143 (47.83%) men; *M*_age_ = 21.87 (SD = 3.23, 16–45 Y); 184 (61.54%) from Asia, 22 (7.36%) from Africa, 66 (22.07%) from Europe, 27 (9.03) other; *M*_LOS_ = 113 (37.79%) <6 M, 41 (13.71%) 7–12 M, 62 (20.74%) 13–18 M, 83 (27.76%) >19 M; 220 (73.58%) single, 79 (26.42%) un single	ASSS-28 (Yu et al., 2014)	BSI	AS was correlated significantly with mental health symptoms (Somatization, obsessive-compulsive, interpersonal sensitivity, depression, anxiety, hostility, phobic anxiety, paranoid ideation and psychoticism) (*r* = 0.458)
Zasiekina and Zhuravlova (2019), Ukraine	Correlational	African international students (Nigeria, Ghana, Zimbabwe, Namibia, Senegal) (*n* = 41), 12 (29%) female, 29 (71%) male; *M*_age_ = 25.14 (SD = 2.6 Y)	ASSIS-36	Procrastination Scale (Lay, 1986)	Acculturative stress was not a significant predictor of depression (*β* = −0.07)

AS, Acculturative Stress; ASSCS, Acculturative Stress Scale for Chinese Students; ASSIS-36, Acculturative Stress Scale for International Students; ASSS-28, Acculturative Stress Scale for Students; BSI, Brief Symptom Inventory; BYAACQ, The Brief Young Adult Alcohol Consequences Questionnaire; CADC, Cultural Adjustment Difficulties Checklist; CAS, The Chinese Affect Scale; CAS1, Career Aspiration Scale; CES-D, Center for Epidemiologic Studies-Depression Scale; HSC, Hopkins Symptom Checklist (Psychological Distress); ILS, Index of Life Stress; MASI, Multidimensional Acculturative Stress Inventory; MCS, Mental Health Component Summary; MDQ, Menstrual Distress Questionnaire; MHPLP, Modified Version of the Health Promotion Lifestyle Profile; MOS (SF-12), Medical Outcomes Study Short Forms; MTF, Monitoring the Future Survey; OHRQo, Oral Health-Related Quality Of Life; OQ, Outcome Questionnaire 10.2 (five items of this scale were used to measure psychological distress); PANAS, Positive and Negative Affect Scale; PHQ-9, Patient Health Questionnaire Depression Module; PIA, Perceived Intergroup Animosity; PMS, Premenstrual Symptoms; POH, Perceived Out-Group Hostility; PSS, Perceived Stress Scale-Short form; SACQ, Student Adaptation to College Questionnaire; SOC, Sense of Coherence; SOS-10, 10-item Schwartz Outcome Scale; SWLS, Satisfaction with Life Scale; VOER, Vocational Outcome Expectations Revised.

### Risk of bias assessment

Table 1 provides a complete description of the bias assessment of the studies included. The results reported in this table are obtained from examining the characteristics of the studies included based on the criteria presented in the ARHQ scale. Besides, in Figure 2, the degree of bias of each study based on ARHQ is shown graphically. The first graph in Figure 2 shows the degree of bias of each study, and the second graph shows the results of the overall quality score of the studies included for each of the 11 criteria in the ARHQ. Based on the evaluation, it seems that most of the criteria in ARHQ have been met in the studies. However, the criterion of “Outcome assessment blind to exposure” has not been done in most studies, which can reduce the quality of studies to some extent and increase the type I error in some way. In addition, the two criteria of “sample size calculation” and “analysis controls for confounding” have been observed in almost only half of the studies, which may increase the type II error and type I error, respectively. The absence of accurate sample size calculation, lack of analysis controls for confounding variables, and failure to conduct outcome assessment using a person blind to exposure significantly impact the overall quality and credibility of the studies. These shortcomings indicate reduced generalizability, diminished confidence in results, and compromised interpretability and inference. In studies focusing on complex subjects like humans, the consequences of research are significant. This is because research involving humans requires careful attention to controlling factors that could introduce errors in interpretation and subsequent generalizations. Since humans are the subject of these studies and researchers aim to apply findings to other humans, failure to adhere to these controlling factors can lead to confirming incorrect hypotheses and spreading inaccurate information. Therefore, not following these factors can maintain biases and spread inaccurate information about humans.

To ensure adherence to these standards, authors should prioritize thorough planning and consultation with methodological experts and employ rigorous peer review processes. Additionally, ongoing education and training for researchers on best practices in study design and analysis can further enhance the quality and credibility of research outcomes. One approach to ensure adherence to all principles outlined in the study quality control tool is to conduct all studies through a proposal writing process. In this process, proposals undergo rigorous peer review, and upon meeting all outlined criteria, approval for study execution is granted. The three limitations highlighted above can be subject to specific scrutiny during the review process. By adopting this method, researchers can ensure that studies are meticulously planned and executed, thereby enhancing their quality and credibility. Additionally, this approach promotes transparency and accountability in research practices, ultimately contributing to the advancement of knowledge in the field.

Additional information on the degree of bias of each study can be found in Table 1 and Figure 2.

### Measures used in the included studies

All studies included had used valid and standardized questionnaires in the country of study to evaluate the variables. Regarding the variable of AS, most of the studies included, i.e., 21 studies out of 29, have used the ASSIS 36 questionnaire (Sandhu and Asrabadi, 1994) to measure this variable. In other studies, similar questionnaires were usually used to measure the variable of AS. In addition, one of the psychological consequences measured as a dependent variable in the studies included is depression, which in most studies was measured using the CES-D questionnaire (Radloff, 1977). Table 2 lists the names of the questionnaires used in the studies included to measure AS and its psychological consequences.

### The relationship between AS and psychological outcomes

A review of studies on the relationship between AS and its consequences in international students showed that AS has a significant relationship with an increase in significant negative psychological consequences such as depression, psychological distress, etc., as well as a decrease in positive psychological consequences such as psychological adjustment, mental health and so on as described in detail below.

#### The increase of negative psychological consequences

According to the studies included, it seems that the most important negative psychological consequence in international students is depression. Thus, most of the studies included in which the aforementioned relationship was examined showed that there is a significant positive relationship between AS and depression in international students and the correlation coefficient between these two variables ranged from 0.18 to 0.69 (Constantine et al., 2004; Wei et al., 2007; Hamamura and Laird, 2014; Bai, 2016; Liu et al., 2016; Gebregergis et al., 2019, 2020; Cheung et al., 2020). Only one study showed that this relationship is not significant (Zasiekina and Zhuravlova, 2019). Moreover, the relationship between depression, as one of the subscales of mental health syndrome, and AS was examined in two other studies, the results of which indicated a significant positive relationship between them (Kilstoff and Baker, 2006; Yang et al., 2018; Taušová et al., 2019). Therefore, it seems that the higher the AS in international students, the more likely they are to develop depression, one of the most likely negative psychological consequences.

Another common consequence of AS in international students is psychological distress. The correlation between these two variables was examined in three studies included and the correlation coefficient between these two variables ranged from 0.40 to 0.56 (Lee et al., 2004; Wei et al., 2012a,b). Therefore, it can be said that second to depression, psychological distress can also be a negative psychological consequence of AS. Other negative psychological consequences of AS in international students include alcohol abuse (Hunt et al., 2017; Kim and Cronley, 2018), mental health symptoms (Yang et al., 2018; Taušová et al., 2019), negative emotions (Pan et al., 2007), perceived general stress (He et al., 2012), and premenstrual stress (Lee and Im, 2016, 2017).

#### The decrease of positive psychological consequences

In addition to increasing the negative psychological consequences, AS in international students can also show its negative effects by reducing the positive psychological consequences. Looking at the studies included, it is possible to conclude that the most common psychological consequences of AS are the decrease of the psychological adjustment (Yakunina et al., 2013a,b; Lashari et al., 2018) and mental health (Bhandari, 2012; Kim and Cronley, 2018) in international students. Therefore, it seems that the higher the AS in international students, the lower the psychological adjustment and their mental health is the most likely psychological consequence. In addition, based on the rest of the studies, it can be stated that AS can negatively associated with the career outcome expectation (Reynolds and Constantine, 2007; Franco et al., 2019), life satisfaction (Bai, 2016; Taušová et al., 2019), health promotion behaviors (Kim and Yoo, 2016), quality of life (BenGhasheer and Saub, 2020), resilience (Kim and Cronley, 2018), and sense of coherence (He et al., 2012) in international students.

### Meta-analysis

**Figure 3:**
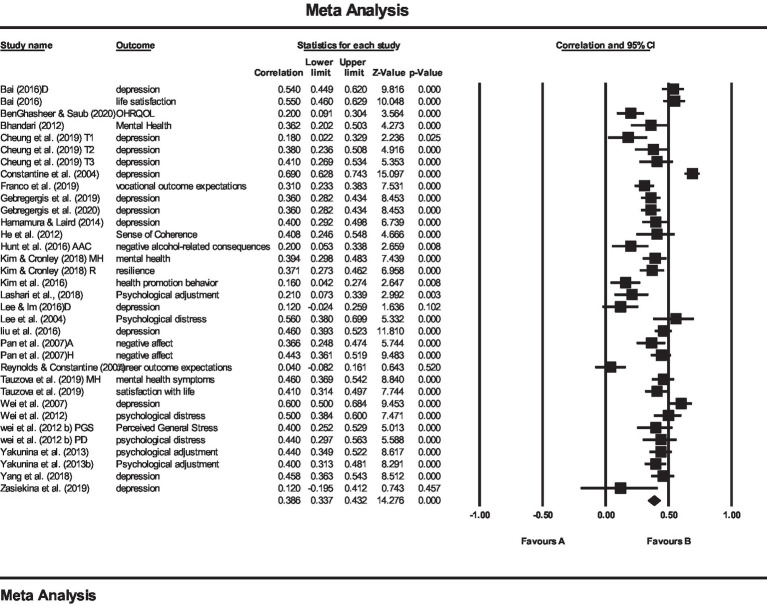
The plot of meta-analysis of the relationship between AS and psychological outcomes. *Q* = 229.729 *p* < 0.001, *I*^2^ = 85.635.

**Figure 4:**
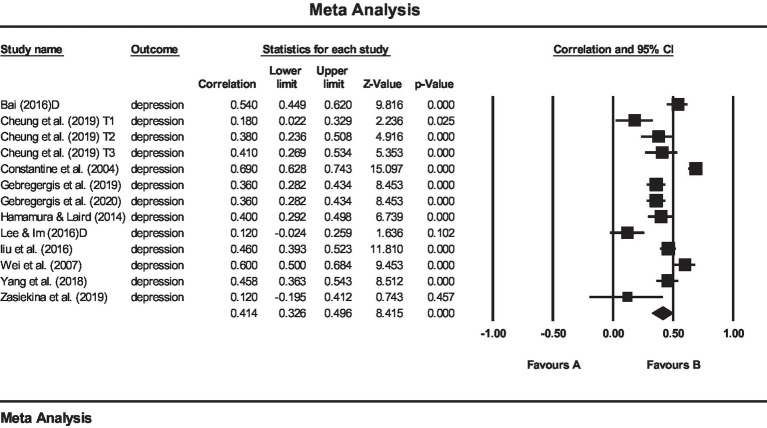
The plot of meta-analysis of the relationship between AS and depression. *Q* = 109.568 *p* < 0.001, *I*^2^ = 89.048.

Two separate meta-analyses were performed to examine (1) the relationship between AS and psychological consequences and (2) the relationship between AS and depression. The results of the first meta-analysis showed that the mean effect size for the relationship between AS and psychological outcomes (like depression, life satisfaction, quality of life, vocational outcome expectations, drinking behaviors, resilience, health promotion behavior, psychological adjustment, psychological distress, negative affect, and mental health symptoms) was *r* = 0.39 (see Figure 3). The results of the second meta-analysis also showed that the effect size for the relationship between AS and depression was *r* = 0.41 (see Figure 4). Both mentioned effect sizes fall into the moderate effect category, according to the Cohen’s (1988) classification, which indicates the very important role of AS in predicting the psychological consequences of international students, one of the most important of which is depression.

In addition, *Q* and *I*^2^ tests were used to examine the heterogeneity of effect sizes. The value of *Q* was significant for all studies equal to 229,729, with a degree of freedom of 35 for the relationship between AS and psychological consequences (*p < 0*.001), which indicates the heterogeneity of effect sizes. Also, the *I*^2^ value for the above relationship was 85.635. A *Q* value of 109,568, with a degree of freedom of 12, showed also a significant relationship between AS and depression, indicating a heterogeneity of effect sizes. Also, the amount of *I*^2^ for the above relationship was 89.048. The value of *Q* at 109.568 was significant for the relationship between AS and depression for the freedom degree 12, indicating the heterogeneity of effect sizes.

**Figure 5:**
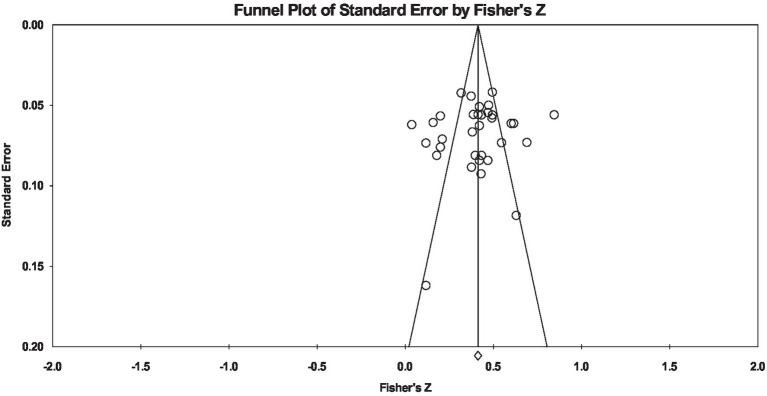
The funnel plot of standard error for assessment of the publication bias of studies included.

The funnel diagram in Figure 5 shows the extent of publication bias in studies included in the meta-analysis. As can be clearly seen in Figure 5, the studies included in this meta-analysis have formed a symmetric distribution on both sides of the effect size line. There are almost no studies on either side of the effect size line that are extremely far from this line, and most studies are located around this line and inside the drawn triangle. The Kendall’s tau value was −0.041, which was not significant in both 1-tailed (*p* = 0.366) and 2-tailed (*p* = 0.733) statuses. Egger’s test was also used to check the symmetry of the funnel diagram. Egger’s regression intercept value was −0.89, which were not significant in 1-tailed (*p* = 0.315) and 2-tailed (*p* = 0.630) statuses. According to the two indicators above, it seems that there is no publication bias in the studies included.

## Discussion

This systematic review including two meta-analyses was conducted to investigate the relationship between AS and psychological consequences in international students. The consideration of the studies included illustrated that AS is one of the effective variables in predicting the increase in important negative psychological consequences such as depression, psychological distress, alcohol abuse, mental health symptoms, negative emotions, perceived general stress, premenstrual stress, and prediction of reducing important psychological outcomes such as psychological adjustment, mental health, career outcome expectation, life satisfaction, health-promoting behaviors, quality of life, resilience and a sense of coherence. These findings were also confirmed in the meta-analyses performed in that it was found that AS was associated with psychological consequences (*r* = 0.38), and with depression in international students (*r* = 0.41).

In interpreting the findings of this systematic review and meta-analysis that the AS in college students is related to their psychological outcomes, and the significant effect size obtained, especially for depression, we can refer again to Berry’s (2006) AS model. According to this model, the stage after experiencing stressors, in which a person evaluates the amount of stress ahead and his mental sources to deal with this stress, is the stage of experiencing stress. Therefore, it can be said that people who do not have sufficient mental sources to deal with the AS, in the gap between experiencing stress and reaching relative adaptation, suffer from an erosive stress that reduces their mental energy and prepares the ground for suffering from psychological disorders. The interesting point to note is that the variables mentioned this AS model also include anxiety, depression, and psychosomatic disorders, which are also clearly visible in our findings. To the extent that depression was found to be the most frequent psychological outcome in our systematic review, a separate meta-analysis was performed for it, which demonstrated a significant effect size.

Given that in some studies included the relationship between AS and psychological consequences has been studied as a structural model, in addition to explanations based on models and conceptual theories in this field of inquiry, the role of these mediating variables in the relationship can be also briefly reviewed. Some studies have also pointed out important factors that can control the negative effects of AS, which have also been reviewed.

### How AS results in undesirable psychological consequences

#### Anxiety

He et al. (2012) stated that international students entering a foreign country face challenges such as language skills, adapting to a new education system, and adjusting to life (such as finding a place to live and a part-time job for financial support) experience high levels of anxiety. Although this type of anxiety will decrease in the coming years, students will still be involved in stressful challenges such as finding a new curriculum, and deciding on future plans. Perceived anxiety in students is not limited to this extent and sometimes causes international students to stay away from the community. In this way, these students procrastinate their presence in international gatherings. In this regard, Zasiekina and Zhuravlova (2019) believe that one of the reasons for procrastination, which can have devastating effects on the process of acculturation of students, is the fear of negative evaluation. In this way, students procrastinate the performance of academic tasks and interaction with students and people of the host culture for fear of being negatively evaluated, which can deprive them of the space they need to reduce AS and prevent the reduction of AS in them, which in turn paves the way for negative psychological consequences.

As explained in Berry’s (2006) model, when faced with a new culture and at the beginning of the acculturation process, individuals compare the upcoming stress and their psychological sources. At this stage, if they assess their sources as sufficient to cope with the AS, they will be less likely to experience anxiety, and if they assess their sources as insufficient, they will experience a high level of anxiety. In other words, the comparison of the upcoming task and the available psychological resources determines the level of individuals’ anxiety. Experiencing a high level of anxiety can accelerate the process of people’s psychological exhaustion and make them more vulnerable to other psychological disorders. Therefore, the two factors of anxiety as well as the duration of the anxiety (the time factor mentioned earlier) can be the most important predictors of other psychological consequences, one of the most important of which seems to be depression. In this systematic review, depression has been found as a consequence with a moderate effect size.

#### Depression

When exposed to AS, international students may be reluctant to share their experiences with their peers for fear of embarrassment, as these experiences may indicate their individual failure. In addition, these students may avoid talking to friends or family about their experiences when they return home, as they do not want to burden others with their problems. As a result, carrying these distressing emotions may make these students more vulnerable to depression. International students, on the other hand, usually have outstanding academic performance in their home country and expect to continue to perform well in the host country. But because of a new environment, a different language, and new cultural norms, these students may not be able to maintain the previous quality of their academic performance. The presence of such conditions can contribute to the experience of feeling depressed in these students (Wei et al., 2007).

#### Alcohol abuse

Due to the presence of anxiety and symptoms of depression and psychological distress, students may use non-constructive ways to alleviate their experienced stress, including alcohol use (e.g., Hunt et al., 2017). One of the variables that can play a mediating role in the relationship between AS and psychological consequences is students’ unhealthy behaviors. In a way, during this period, students have problems in terms of nutrition and high-risk behaviors such as smoking and alcohol use that can reduce their quality of life (BenGhasheer and Saub, 2020). Hunt et al. (2017) suggested that international students may use alcohol as a way to cope with AS that leads to more risky behaviors. Although it makes sense to assume that increased alcohol use is likely to increase the consequences of alcohol use, negative ways of coping with AS in students, even when their alcohol use is low, may lead to more risky behaviors. The above can also be confirmed based on Berry (1997), as he has stated that the likelihood of negative consequences of alcohol use can be increased in response to AS and may indicate that the tendency to use alcohol is one of the coping mechanisms in response to the experience of high levels of stress. Particularly, one the vulnerable populations is international students who do not have access to appropriate internal and external adaptive resources (such as social support, a sense of belonging, etc.). These people may turn to maladaptive patterns of stress reduction, such as alcohol use.

### Factors influencing the destructive effects of AS

Although all international students are likely to experience AS, the amount of stress experienced varies based on a number of factors, the most important of which are emotional intelligence, cultural intelligence, social support and language skills. It is worth to be mentioned that there were a number of different variables in the studies included in the current meta-analysis, mentioned as the mediator variables which can buffer the negative effects of AS on international students, among which above-mentioned variables were bolded. Although the frequency of studies in which the above-mentioned variables were examined as a mediator variable between AS and a psychological outcome were not high enough to be included as a mediator variable in the meta-analysis, they were worth enough to be discussed here.

#### Emotional intelligence

People with high emotional intelligence have the ability to regulate their emotions when interacting with others, as well as using their emotions to enhance their psychological adjustment in a different cultural context. They also have a high level of understanding emotions, which enables them to distinguish between the natural flow of emotions and the incorrect expression of emotions. This high understanding of emotions also enables them to be aware of how situation and culture affect the expression of emotions and to be able to understand the emotional aspects of a situation that may be a source of misunderstanding and miscommunication. On the other hand, people with high emotional intelligence have a sensitive and in-depth view of the cultural aspects of expressing emotions, which enables them to effectively modify and reconstruct their emotional patterns in order to perform better in interaction with people from different cultures. Understanding emotions correctly deciphers the underlying causes of individuals’ emotional manifestations, thus facilitating the management of their own and others’ emotions. Cognitive flexibility of people with high emotional intelligence enables them to properly manage irrelevant and potentially interfering responses and reduce conflicts. Therefore, understanding emotions and managing them, by providing emotional flexibility in the network of intercultural interactions of international students, play a very important role in the process of acculturation and promote their intercultural compatibility (Gebregergis et al., 2020).

#### Cultural intelligence

Following Berry’s (2003) AS model and Ang and Van Dyne’s (2008) cultural intelligence model, international students who are aware of the cultural issues of a society before entering that society are more likely to experience less AS. Berry (2003) states that the psychological resources that individuals bring to the field of acculturation will play a very decisive role in their psychological acculturation. Ang and Van Dyne’s (2008) defined cultural intelligence as a mental ability that enables individuals to control and become aware of their own cognitive processes in the face of people from a different culture. Cultural intelligence also enables individuals to critically and consciously modify factors in their culture to better adapt to the host culture. In addition, the ability of these individuals to gain full knowledge and understanding of the cultural practices of the host community enables them to achieve a better cultural understanding that organizes and guides them towards better social interaction in that host culture.

Cultural intelligence helps people in three ways, namely cognitive, motivational and behavioral cultural intelligence. People with high cognitive cultural intelligence perform better in the host culture and form dynamic relationships with people from different cultures. The motivational cultural intelligence of international students makes them eager to acquire important cultural information and learn from students of different cultures. Motivational cultural intelligence also protects individuals by encouraging them to participate in various intercultural experiences of the individual’s internal motivations as a source of personal satisfaction, their external motivations as a source of objective benefits, and their self-efficacy as an opportunity to demonstrate their potentials and capacities. Behavioral cultural intelligence, on the other hand, helps individuals to display appropriate verbal and nonverbal behaviors during their socio-cultural interaction with individuals from different cultural contexts (Ang and Van Dyne, 2008). In fact, behavioral cultural intelligence is the most important aspect of cultural intelligence because it enables people to achieve a sense of control and regulate social behaviors, with the least amount of misunderstanding and attributional problems, in a multicultural context (Gebregergis et al., 2019). In this regard, Yakunina et al. (2013a,b) stated that the universal-diverse orientation of international students is closely related to intercultural experiences and openness to cultural differences that can reduce the mental experience of AS. In addition, students with high scores in diverse global orientations are usually better able to discuss the conflicts of cultural values that occur between indigenous and host cultures. This will reduce the stress of students’ AS and increase their positive intercultural compatibility.

#### Social support

Difficulty in regulating emotions can cause people who experience high levels of AS to begin to distance themselves from those around them in the host country, especially because interacting with these individuals may result in different types of AS (such as language restrictions, value clashes across culture, role changes, and discrimination) in them. For international students, people from the host culture, whether other international students or natives of that culture, can provide good support in finding job opportunities, recognizing cultural differences in the workplace, writing a resume, as well as psychological problems. Each of these reasons is sufficient to explain why social support is important in the host culture in creating positive psychological consequences for international students (Franco et al., 2019).

#### Language skills

Language skills seem to be one of the most important aspects of adapting to a new culture. Thus, relying on the same language speaking friends for social and emotional support may reinforce students’ feelings of shame and inadequacy in dealing with issues that arise in their second language context. Such a lack of language skills and self-confidence may create a negative cycle that prevents international students from seeking academic and social support from people with a host culture. Thus, in the face of academic homework, these students experience more stress in writing an article or presenting an article in the language of the host culture, which in turn increases their feelings of inadequacy, shame, and inferiority (Hamamura and Laird, 2014). Students who think they have poor language skills are less likely to collaborate with intercultural and general counseling groups. Thus, these people think that these advisory groups have little role in guiding and supporting them in regulating the disturbed emotions caused by AS. Hence, they usually rely on themselves and use their willpower to overcome problems. Logically, as a result, these people may experience more stress, which makes them more vulnerable to psychological problems.

### Strengths, limitations and recommendations for future research

The current systematic review possesses several notable strengths, among which the following can be highlighted: (1) Prior to conducting this systematic review, a protocol was prepared and registered in the OSF database. (2) The introduction of this review describes the existing models in the field and elucidated the necessity of their implementation. (3) The search for sources was extensive, encompassing all reputable databases in the field, increasing the likelihood of capturing relevant studies. (4) The inclusion criteria were clearly articulated and based on specific keywords and concepts related to acculturative stress and psychological outcomes in international students. (5) The quality and bias of all included studies were assessed using a validated tool. (6) A meta-analysis was performed on the included studies, providing a clear effect size for the relationship between acculturation stress and psychological outcomes. (7) The discussion extensively delved into interpreting the relationship between AS and psychological outcomes and also addressed the mediating variables in this domain.

When interpreting the results, various limitations must be considered. (1) One of the important issues in this systematic review was that all the studies included in this study had a descriptive design and, in our searches, there were no intervention studies that could be included in this review, why we only express its conclusion about the “relationship” of AS with psychological outputs but it cannot speak of “effect” or “effectiveness.” (2) Another very important issue in this regard is the use of self-report tools in all studies included. In other words, all this information has been collected through questionnaires and in none of the studies, qualitative or mixed information has been provided in order to examine students’ narratives about AS and its psychological consequences for them. (3) The process of converting Beta and Eta values to Pearson’s *r* using an online calculator may introduce potential errors or uncertainties in the effect size estimation. (4) The diverse demographic characteristics of the included studies’ participants, such as different countries, races, and educational backgrounds, may limit the generalizability of the findings to a broader international student population. (5) As mentioned in the results of bias assessments, the absence of accurate sample size calculation, lack of analysis controls for confounding variables, and failure to conduct outcome assessment using a person blind to exposure significantly impact the overall quality and credibility of the studies. These shortcomings reduce generalizability and compromise interpretability and inference.

Given the considerable body of descriptive studies and the conducted systematic reviews and meta-analyses in the field, it is proposed that future studies move beyond and advance towards the design of multivariable structural models. These models can shed light on predictive and outcome variables of AS at the same time within a comprehensive framework. Specifically, it needs to be clarified which variables, such as attachment styles, personality traits, early maladaptive schemas, among others, could serve as stronger predictors for AS, and simultaneously, which variables could be outcomes of AS when AS is positioned as a mediating variable. Furthermore, when AS is considered as a predictor variable in the model, which variables such as self-esteem, social support, stress coping styles, emotion regulation styles, and others, could mediate the relationship between AS and important psychological outcomes like depression, distress and psychological symptoms. Limited studies in this area make it difficult to investigate the role of mediating variables in meta-analyses. It is also advisable for researchers to explore the effectiveness of various psychological interventions on reducing AS among international students to determine which therapeutic interventions are most effective in this area. Conducting structural models and interventional studies in this field of inquiry paves the way for more systematic reviews and meta-analyses, which in turn provide valuable summaries for researchers and therapists working in this field.

### Clinical and practical implications

Several clinical and practical implications of the study result relevant to mental health professionals, educators, policymakers, and other stakeholders working with international students can be suggested. First, *time* is a major factor between experiencing stress and reaching the adaptation stage. Therefore, it is recommended that special implementation programs be considered for this time gap in international students. It seems that implementing psychological training, creating social support, and teaching useful coping styles along with intergroup contact to international students based on Berry’s (2006) AS model can reduce this harmful gap for students and prevent the acculturation process from becoming an erosive process, which in turn reduces psychological damage.

Second, one treatment method based on new ways to reduce health risk behaviors in international students is to increase their resilience. Preventive strategies focusing on resilience not only seek to reduce the negative consequences, but also to improve the positive aspects that highlight a particular source of support in a particular population. Improving the strengths of individuals in a particular class not only increases self-efficacy and self-adequacy, but also fosters the investment process of these supportive factors in creating positive environmental change. Thus, intervention-based and resilience-focused preventive strategies might not only reduce behaviors such as alcohol use among young people, but also create a wave of health-promoting behaviors on student campuses, where peer influence is an important trigger for high-risk behaviors (Kim and Cronley, 2018). Therefore, decision-makers in the field of international students are advised to have pre-determined intervention packages for working with international students so that they can familiarize these students with this concept, its implications and consequences at the beginning of their arrival, and take steps to reduce the negative effects of these conditions on students. In line with this goal, examining the effectiveness of different therapies on reducing AS can also have valuable achievements.

Third, as one of the aspects of sociocultural adaptation in the Berry and Sam (1997) model included the intergroup contact, conducting group interventions by host countries for international students is likely to have positive effects on reducing their acculturation stress. To the extent that Berry and Sam suggested, gaining cultural knowledge, social skills, and language proficiency in the host culture, along with interpersonal and intergroup contacts, can help individuals achieve better adaptation to their new environment. According to the Berry’s model, the most effective acculturation style that will also be associated with lower acculturation stress is the integration, in which immigrants are encouraged to maintain their culture of origin while simultaneously establishing social connections with various ethnocultural groups to enhance intergroup harmony, social cohesion and well-being (Berry et al., 2022).

Following Ward (2008) who believes the application of cultural learning/social skills approach is essential for explaining, predicting, and managing acculturation-related issues, authorities in universities are recommended that topics such as host country culture education for international students, social skills training through workshops, and the establishment of task forces at universities to carry out these activities and prepare students for life in the host culture can significantly aid in their adaptation to the host country’s conditions.

In this regard, Canada, one the pioneers in the policy of multiculturalism (Berry, 2013), has launched a program called the *Youth Perspectives Survey* to identify ways to promote positive intergroup contact (Kaufmann, 2021). It seems that, based on the experiences of Canada and England, developing intergroup contact protocols for international students can have a positive impact on reducing their acculturation stress. In this protocol, elements such as cultural education (raising awareness of differences and cultural histories among groups for better environmental understanding), social activities (encouraging participation in various intergroup social groups and activities to strengthen intergroup contact), cultural counseling (providing cultural counseling services by specialized counselors to improve the psychological well-being and adaptation of international students), joint cultural events (organizing cultural events), communication skills training (enhancing communication and interaction skills for interacting with different groups), assessment and follow-up (conducting evaluations of educational courses and counseling services to determine their effectiveness and implementing appropriate changes), networking (encouraging the creation of communication networks with students from different countries for sharing experiences and enhancing intergroup contact), stress management (establishing stress and coping management programs) can be included. To confirm the effectiveness of intergroup contact, Ye (2006) has also clearly demonstrated that students who participated in online interindividual interactions perceived less discrimination, experienced fewer negative emotions, and, overall, had lower acculturation stress.

## Data availability statement

The original contributions presented in the study are included in the article/supplementary material, further inquiries can be directed to the corresponding author.

## Author contributions

RS: Writing – original draft, Visualization, Software, Resources, Methodology, Investigation, Formal analysis, Conceptualization. MM: Writing – review & editing, Writing – original draft, Visualization, Software, Resources, Methodology, Formal analysis, Conceptualization. SF: Writing – review & editing, Validation, Supervision, Resources, Project administration, Methodology, Investigation, Conceptualization.

## Footnotes

^1^ https://osf.io/6ajcw/?view_only=e7a02547f341464f951fcb4dc23973dc

^2^ psychometrica.de
